# A deep learning approach to assess transendothelial cell trafficking performance

**DOI:** 10.1038/s41598-026-46045-4

**Published:** 2026-04-03

**Authors:** Thomas Michael Schumacher, Elisabeth Marie Gottloeber, Eric Koziel, Mehmet Sacma, Kaya Eichhorn, Luana Raiber, Johann Gout, Jessica Lindenmayer, Elodie Roger, Michael Karl Melzer, Hartmut Geiger, Patrick Christian Hermann, Ninel Azoitei, Thomas Seufferlein, Alexander Kleger, Reinhold Schirmbeck, Medhanie Assmelash Mulaw, Yazid Josef Resheq

**Affiliations:** 1 Department of Internal Medicine I, Ulm University Hospital, 89081 Ulm, Germany; 2 Institute for Molecular Oncology and Stem Cell Biology, Ulm University Hospital, 89081 Ulm, Germany; 3 Unit for Single-Cell Genomics, Ulm University, 89081 Ulm, Germany; 4 Department of Urology, Ulm University Hospital, 89081 Ulm, Germany; 5 Core Facility Organoids, Ulm University, 89081 Ulm, Germany; 6 Institute of Molecular Medicine, Ulm University, 89081 Ulm, Germany; 7 Division of Interdisciplinary Pancreatology, Department of Internal Medicine 1, Ulm University Hospital, 89081 Ulm, Germany

**Keywords:** Biological techniques, Cancer, Computational biology and bioinformatics, Engineering

## Abstract

Transendothelial migration (TEM), is a complex, multistep process impacted by diseases like autoimmune disorders and cancer. Deciphering aspects of the process enhances our understanding and possibilities for disease treatments. The extent to which this potential can be leveraged is often limited due to conventional assays neither accurately mimicking specific *in vivo* conditions like sheer stress nor visualizing the whole transmigration cascade, hence missing distinct mechanisms. Flow-based adhesion assays overcome these limitations and allow the use of various endothelial cells from different tissues with distinct properties. So far, a broader and translational assay application is hampered by potential operator-based bias/lack of standardization, as well as poor scalability due to time-consuming manual analysis. In this study, we successfully combined this assay with AI-based analysis including subsequent classification of cell-transmigration phases by a Keras/TensorFlow-based deep learning model. Trained on healthy-donor and pancreatic cancer patient-derived T cells, the model achieved a high accuracy of 91.6 % in identifying/categorizing cell transmigration, surpassing the currently accepted 80 %-threshold, therefore qualifying as a fast, standardized AI-based live-cell imaging tool. Additionally, its architecture grants highly convenient reconfiguration for various disease-model investigations. Hence, by combining an affordable and simplistic, yet potent live-cell-imaging technique with a comprehensive AI-approach, we have established a powerful tool which allows for integrating TEM-assays into various disease models.

**Supplementary Information:**

The online version contains supplementary material available at 10.1038/s41598-026-46045-4.

## Introduction

The human immune system serves as a prime example of the importance of the cell-extravasation-cascade, but is not limited to it, as cell extravasation also plays a crucial role in cancer metastasis^1^. The ability of the immune system to respond rapidly and accurately to sites of injury, infection and malignancy relies fundamentally on the migration of immune cells across cell barriers to detect and subsequently eliminate pathogens, as well as the involvement in tissue-repair and -homeostasis^2^. Deciphering this process and its individual steps further enhances our understanding and the possibilities of developments for treating various medical conditions. Migration to the target regions occurs through a complex transmigration cascade. The process of transendothelial migration (TEM) is a fundamental physiological mechanism encompassing the exit of immune cells from the bloodstream and their subsequent migration into tissue. This tightly regulated, multistep cascade unfolds in a distinct sequence of phases, including the capture, rolling, arrest, intravascular crawling, and paracellular or transcellular transendothelial migration^3^. The extravasation is mediated through different pathways and a complex interplay of chemokines, integrins, selectins and kinases, depending on the vascular bed, the type of leucocyte and the inflammatory signaling. As a common ground, this process is initiated through the detection of chemokine gradients (e.g. CXCL8) released from inflamed tissues by the addressed immune cells. The chemokine gradients activate G protein-coupled receptors on these immune cells, which in turn triggers signaling enhancing integrin-affinity. Focal adhesion kinase (FAK) and the PI3K/Akt pathway are central in this first step. During the rolling step, immune cells engage in selectin-mediated (e.g. L- and E-/P-selectin) low-affinity interactions upon reaching the apical side of the endothelium. Firm adhesion is enabled through the binding to endothelial ligands (e.g. Intercellular adhesion molecule 1 (ICAM-1) and Vascular cell adhesion molecule 1 (VCAM)) after conformal changes of integrins (e.g. lymphocyte function-associated antigen-1 (LFA-1)) triggered by chemokines. Crawling along the endothelium is then, amongst others, dependent on integrin ICAM-1/LFA-1 interactions and continued FAK signaling. The crossing of the endothelium on para- or transcellular routes involves molecules like the Platelet and endothelial cell adhesion molecule 1 (PECAM- 1) and vascular endothelial cadherin (VE-cadherin)^3–7^. In some cases, specific adhesion molecules may be involved, such as MADCAM-1 which guides immune cells into inflamed mucosal tissue within the intestine. Such specific adhesion molecules may then be exploited for treatment of diseases such as the application of the mAB Vedolizumab in the treatment of Colitis Ulcerosa^5^. Disruptions in this fine-tuned process can lead to significant pathological consequences. While an impaired extravasation might result in an inadequate response to tumors or infections, an excessive or misdirected leucocyte migration might drive chronic inflammation, most notably autoimmune diseases^8^.

Pancreatic ductal adenocarcinoma (PDAC) is one of the diseases which is characterized by a strongly impaired anti-tumor immune response. Among neoplasms of the human pancreas, PDAC is considered the most common malignancy with a particular poor prognosis compared to other solid tumors^9^. 90 % of PDAC cases are diagnosed at an advanced, in many cases incurable, stage as clinical symptoms usually manifest after the tumor has metastasized to non-loco-regional such as the liver^10,11^. Noteworthy, PDAC exhibits high resistance to antineoplastic therapy and its tumor-microenvironment (TME) is described as an “immune desert” preventing immune cells from infiltrating,  resulting in limited treatment options^12^. The TME, where cancer cells are imbedded in, plays a central role in growth and metastatic potential of the tumor as well as a determinant of resistance to treatment. TME in PDAC factors in its primary and acquired resistance to antineoplastic treatment approaches. This is not limited to chemotherapy but also involves targeted and immune-modulating treatments^13^.

In 90 % of the PDAC cases the Kirsten rat sarcoma virus (KRAS) oncogene is mutated. The most common KRAS mutations in PDAC are G12D (44 %), G12V (34 %) and G12R (20 %)^14^. Patients with KRAS mutations (especially G12D and G12V^15^ have a particularly poor prognosis due to, among others, more aggressively invasive metastasis, changes to the TME and reduced therapy response^16,17^.

Furthermore, parts of the innate and adaptive immunity are adversely affected by ageing, which results in a general decline of immune competence. In part this is due to a constant deterioration in *de-novo* production of naïve T cells, restriction of T cell receptor (TCR) repertoire and increasingly impaired activation of T cells^18^. Additionally, these effects seem to be different between male and female individuals as, for example, the proliferative capacity of T cells, the number of regulatory T cells (Tregs) and the CD4/CD8 ratio differ^19–21^.

Studying the extravasation phases in the laboratory is highly challenging and has traditionally relied on reductionist approaches. Current common *in vitro* migration assays, such as Transwell^®^, allowing quantification of directed migration across a membrane, fail to accurately replicate these processes: By design, key physiological factors, most notably shear stress, as this assay largely relies on gravity from a physical perspective. While *in vivo* mouse models provide a more physiologically relevant and complex environment, they do not fully reflect either the human innate nor the adaptive immune system (like the difference of the balance of lymphocytes and neutrophils, tyrosine kinase receptor expression on hemopoietic stem cells or the expression of defensins^22^. Furthermore, they are highly resource- and cost-intensive, causing limited scalability^22,23^.

Flow-based adhesion assays offer a valuable alternative for the analysis of individual extravasation phases. Originally, these assays were developed for the visualization of lymphocyte recruitment across hepatic sinusoidal endothelial cells^24^. These assays are not only applicable to immune cells, like lymphocytes and neutrophils, but also extend to the study of tumor cell behavior, particularly the migration of highly motile cells like melanoma^25^. The microcapillary slides can be used to grow endothelial cells from every organ or even from tumors, especially important for investigations of processes in distinct organs such as the liver featuring highly permeable fenestrated sinusoids (small, irregularly shaped, blood vessels found in certain organs, especially the liver) in contrast to other vascular beddings^26^. By simulating physiological shear stress and enabling real-time visualization of cellular interactions with the endothelium, these setups allow the dissection of specific steps of the extravasation cascade. This is crucial for furthering our understanding of vital processes of the cell extravasation cascade, in particular TEM^27^.

However, despite the methodological advantages, several limitations restrict broader applicability. A significant drawback is the susceptibility to operator bias and the limited capacity to analyze meta findings like cell clustering. Additionally, the labor-intense and time-consuming manual analysis presents a bottleneck, which impacts scalability and precludes timely analysis.

To assess these challenges and enhance the assessment of T-cell transmigration, we introduced a novel approach that integrates a modified flow-based adhesion assay^24^ with confocal microscopy and subsequent analysis using a deep learning model based on an R-implementation of Keras/TensorFlow. This model was developed and trained to provide a standardized and fast analysis tool for our flow-based adhesion assay, enabling a comprehensive assessment of immune cell extravasation. In doing so, we aimed at deeper insights into (disease) mechanisms, e.g. in cancer, autoimmunity or age-related changes of the immune system potentially enabling advances in immune-based therapeutic strategies in the future.

## Methods

### Ethics declaration and study protocol

The study was approved by the Ethics Committee (230/14 and 72/19) of Ulm University, Germany, and conducted in accordance with the Helsinki Declaration. All donors/patients provided written informed consent prior to acquisition of blood or tissue. Healthy-donor T cells were isolated from buffy coats provided by the German Red Cross (DRK-KV Ulm e.V., Ulm, Germany) from regular blood donations. Patient T cells were isolated from frozen PBMC samples provided by the biobank of the University Hospital of Ulm, Ulm, Germany.

### Isolation and activation of T cells

First, peripheral blood mononuclear cells (PBMCs) were isolated using Lymphoprep (Stemcell Technologies, Cologne, Germany, #07851/07861) according to the manufacturer’s density gradient centrifugation protocol. Adjunct, pan-T cells isolation was performed with Dynabeads Untouched Human T Cells (ThermoFisher Scientific, Sindelfingen, Germany, #11344D) in accordance with the manufacturer’s protocol. Afterwards, T cells were frozen for storage to ensure comparable conditions as for patient samples. 48 h prior to the experiment the T cells were thawed and incubated for 24 h at 37 °C, following activation by employing Dynabeads human T-Activator CD3/CD28 (ThermoFisher Scientific, Sindelfingen, Germany, #11161D) for 24 h at 37 °C in T cell medium (94 % RPMI Medium 1640 (1×) (Gibco, ThermoFisher Scientific, Paisley, Scotland, #31870-025), 10 % heat inactivated FBS (Gibco, ThermoFisher Scientific, Grand Island, NY, USA, #16140-071), 1 % 10,000 Unit/mL Penicillin/Streptomycin (Gibco, ThermoFisher Scientific, Grand Island, NY, USA, #15140-122). Patient T cells were isolated and activated from frozen PBMC samples using the same protocol.

### Flow-based adhesion assay

The flow-based adhesion assay was adapted from ref.^24^ and the execution will be described below.

The basis of the assay is the visualization of cell interactions/to analyze adhesion (e.g. leucocyte recruitment) under conditions of flow to simulate physiological conditions (e.g. shear stress, here: 0.05 Pa^24^). Detailed experimental assay setup and execution will be presented below in the corresponding section. In brief, cell monolayers (i.e. endothelial cells), cultivated in the channels of microcapillary slides (collagen IV-coated Ibidi µ-slides), are perfused under laminar flow between the two surfaces of the channels to engage “*in vivo*-like” TEM and visualize the adhesion cascade of cells (e.g. immune cells onto the endothelial layer) on a microscopic level^24^. Mimicking *in vivo*-sheer stress, generated by the perfusion, is crucial as various interactions of the adhesive process only occur under these conditions^28,29^.

### Seeding of cells in µ-slide

Each channel of the collagen IV-coated µ-slide (ibidi, Graefelfing, Germany, #80602) was washed using 100 µL DPBS (Gibco, ThermoFisher Scientific, Grand Island, NY, USA, #14190-094). Prior to seeding into the microcapillary slides, Human Umbilical Vein Endothelial Cells (HUVEC) (ATCC, Manassas, VA, USA, #CRL-1730) were cultivated in a T175 flask until confluence and retrieved using Trypsin-EDTA (PAN-Biotech GmbH, Aidenbach, Germany, #P10-023100), washed in DPBS and resuspended at 1 × 10^6^ cells/mL in Endothelial Cell Growth Medium 2 (PromoCell, Heidelberg, Germany, #C-22022). 100 µL of this cell suspension was then added to each channel of the µ-slide. The cells were incubated at 37 °C with 5 % CO_2_ in an incubator for 2 h ensuring cell adherence followed by replacement with 100 µL fresh medium. The endothelium containing microcapillary slides were then incubated at 37 °C with 5 % CO_2_ in an incubator for 24 h to reach confluence. The confluency level was cross-checked prior to stimulating with TNF-α and every flow-based adhesion assay to ensure that confluency reached at least 95 % (as shown in Supplementary Fig. 1a).

### TNF-α stimulation of cells in µ-slide

To ensure relevant expression of adhesion molecules through inflammatory signaling, the endothelial monolayer was stimulated employing TNFα. Cell growth in the µ-slide was assessed after 24 h incubation under an inverted phase contrast microscope (DMIL, Leica, Wetzlar, Germany). Each channel was washed by adding and removing 100 µL of Endothelial Cell Growth Medium 2. This growth medium was then replaced with growth medium containing TNF-α (Gibco, ThermoFisher Scientific, Grand Island, NY, USA, #PHC3011) at 10 ng/ml following incubation for 24 h at 37 °C with 5 % CO_2_ prior to flow-assay. The TNF-α containing medium was replaced with growth medium immediately prior to the experiment, also serving to remove debris.

### Fluorescence staining of T cells and HUVEC in µ-slide

Immediately before the experiment, T cells were stained with CellTrace™ CFSE (Invitrogen, ThermoFisher Scientific, Carlsbad, CA, US, #C34570). HUVEC cells in the µ-slide were counterstained with CellTrace™ Far Red (Invitrogen, ThermoFisher Scientific, Carlsbad, CA, US, #C34572) according to the manufacturer’s protocol.

### Experimental setup

For the first model we employed the Plan Apo 20×/0.8 objective (Carl Zeiss Microscopy Deutschland GmbH, Oberkochen, Germany) mounted on a Zeiss Axioscope2 microscope (Carl Zeiss Microscopy Deutschland GmbH, Oberkochen, Germany) equipped with an Axiocam 702 and ZEN3.1 imaging software (Carl Zeiss, Oberkochen, Germany). The second model used the Zeiss LSM 710 inverse confocal microscope (Carl Zeiss Microscopy Deutschland GmbH, Oberkochen, Germany), equipped with ZEN3.1 imaging software (Carl Zeiss Microscopy Deutschland GmbH, Oberkochen, Germany). Both devices were equipped with an incubation chamber (Carl Zeiss Microscopy Deutschland GmbH, Oberkochen, Germany) to maintain an optimal temperature of 37 °C at 5 % CO_2_.

Two 10 mL syringes (B. Braun Melsungen AG, Melsungen, Germany, #4606108V) were attached, after removal of their plungers, to the two in-flow ports of a medical 3-way valve (B. Braun Melsungen AG, Melsungen, Germany, #16495 C). One syringe was filled with cell free T cell medium (see above) and the other with T cell solution (0.5 Mio. T cells/mL medium). 25 cm of silicone tubing (Ibidi, Graefelfing, Germany, #10841) were attached to the out-flow port and the efferent end connected to the afferent side of the first channel of the µ-slide after filling of the tubing with T cell medium to flush out air bubbles, using an elbow connector (Ibidi, Graefelfing, Germany, #10802).

An injection line (Fresenius Kabi AG, Bad Homburg, Germany, #9004132) had one connector removed and was attached to the port of a 50 mL glass syringe (Carl Roth GmbH&Co. KG, Karlsruhe, Germany, #C683.1) using 1 cm of silicone tubing (ibidi, Graefelfing, Germany, #10841). The glass syringe was placed in a programmable retraction perfursor (Harvard Apparatus, Holliston, MA, USA, #70-3007) and the injection line connected to the efferent side of the first channel of the µ-slide using an elbow connector (ibidi, Graefelfing, Germany, #10802), after the line was filled with cell-free T cell medium to avoid air bubbles entering the system.

### Assay procedure

The channel was perfused for 5 min with the T cell solution by employing a programmable retraction perfusor (Harvard Apparatus, Holliston, MA, USA, #70-3007) at a constant rate of 0.28 mL/min (equivalent to a shear stress of 0.05 Pa) as described in Ref.^30^. Then the 3-way valve was switched to the cell-free T cell medium for 3 min at the same flow rate.

The Z-stack was calibrated to the thickness of the endothelial layer. Using the phase contrast microscope, we selected 12 representative high-power fields (HPF; 200× total magnification) along the channel which were then automatically imaged in the opposite direction of flow.

Adjunct, the tubing was reconnected to the second channel while avoiding air bubbles entering the system. The T cell solution was taken out of the syringe connected to the valve and replaced with a T cell solution from a different donor.

The 12 defined high-power-fields were manually analyzed by two independent operators in a blinded manner. In case of discordance greater than 10 %, a third operator re-analyzed the corresponding experiment. The average of the counted values was then employed to evaluate the accuracy of the AI-based analysis.

### Keras/TensorFlow-based deep learning

An R implementation of the Keras/TensorFlow (V2.15) binary classifier was used for the deep learning analysis. An initial layer with an additional three hidden layers was used, with a final output layer of one binary unit. The model was trained by sampling data from each parameter (healthy donor-derived, PDAC patient-derived (KRAS^G12D^ /KRAS^WT^) 2438 T cells). A total of 2541 T cells were put aside as a test set for validating the accuracy of the model.

### Statistical analysis

Each experiment was tested with a minimum of three biological replicates. All statistical analyses were performed using GraphPad Prism 8.0.1 (GraphPad Software Inc., La Jolla, CA, USA). The number of biological repeats (*n*), included in the final statistical analysis, is indicated in each figure legend. Comparisons between multiple groups were performed using one-way analysis of variance (ANOVA), followed by Tukey’s post hoc test for multiple comparisons. Statistical significance values are reported as *P ≤ 0.05, **P ≤ 0.01, ***P ≤ 0.001, ****P ≤ 0.0001.

## Results

### Modification and improvement of the flow-based adhesion assay

**Fig. 1:**
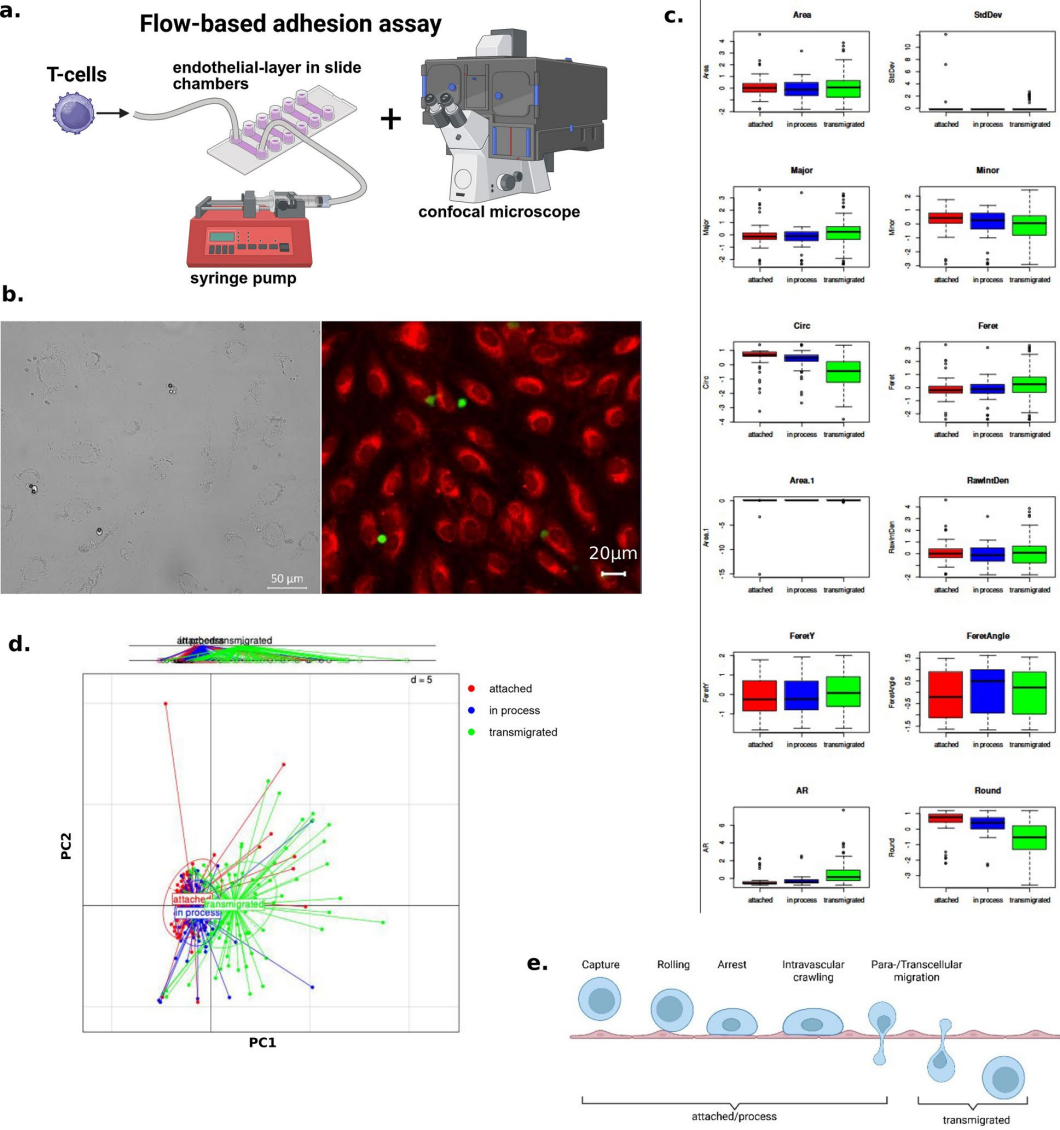
(**a**) Schematic representation of the improved flow-based adhesion assay by incorporation of confocal microscopy. Isolated Tcells are perfused through a chamber slide (using a syringe pump) over a layer of HUVEC cells. The cell transmigration is observed and recorded by confocal microscopy and subsequently analyzed through a trained deep learning system. (**b**) Comparison of microscopy recordings employing phase contrast microscope used in the original setup (left) and the confocal microscope used in the improved model (right). Red = Endothelial cells (HUVEC); green = T cells. (**c**) Geometric feature variance in filtered microscopy images. Area, Area; StdDev, Standard Deviation; ; Major, Major Axis Length; Minor, Minor Axis Length; Circ, Circularity; Feret, Feret Diameter; RawIntDen, Raw Integrated Density; AR, Aspect Ratio; Round, Roundness. (**d**) BGA plot showcasing a lack of segmentation between the features of the transmigration phases. (**e**) Transmigration schematic showcasing the grouping of the several extravasation cascade steps for the training of the system. Created with BioRender.

The initially established flow-based adhesion assay^24^ relies on the perfusion of cells over an endothelial monolayer in a commercial microcapillary channel slide developed for immunofluorescence assays and live-cell-imaging under either static or flow conditions. This chamber is equipped with an afferent and efferent conus allowing the continuous perfusion of fluids such as cell suspensions at a certain rate which in turn exerts a specific sheer stress in the inner part of the chamber (hereafter channel) (Fig. 1a). For further information see Ibidi.com (µ-Slide VI 0.4, #80606). This setup initially utilized phase contrast microscopy to visualize and record the migration of the transmigrating cells across the endothelial monolayer within the microcapillary slides and relied on manual identification and (TEM phase-) classification of the captured cells. Throughout the reported study here, we modified this setup employing confocal microscopy (Fig. 1b) for the reasons described below. Using these microscopy images for the deep learning training to automate the analysis, we faced significant challenges for the deep learning system to distinguish between T cells and confounding image elements, such as smaller air bubbles and cell debris, and in subsequently classifying migration stages of the T cells migrating through the endothelial cells accurately (as shown by the BGA plot and geometric feature variance in Fig. 1c,d). The lack of discrimination between T cells and other image structures presented a central obstacle in reaching a high identification accuracy. The similarity in geometric features through this lack of clear separation obstructed the training to such an extent that the rate of correctly identified and classified T cells remained not suitable for standardization of the analysis.

ImageJ-based preprocessing (background subtraction, noise reduction, and contrast normalization) was used as an initial step prior to DL-based classification. We additionally explored classical thresholding- and morphology-based segmentation pipelines implemented in ImageJ during preliminary analyses. However, these approaches proved insufficiently robust for reliable classification of transmigrating versus non-transmigrating T cells due to heterogeneous fluorescence intensity, overlapping cells, and variable cell morphology under flow conditions.

Reaching the limits of traditional phase contrast microscopy, we incorporated fluorescence staining of the endothelial cells (HUVEC) and T cells used in the assay allowing for visualization by employing confocal microscopy (as shown and previously mentioned in Fig. 1a,b).

In our experiments, we were interested in qualitative data (cells labelled or not), and labelling intensity primarily varied between independent experiments but was generally very bright.

To ensure consistent image quality between experiments/replicas we therefore manually adjusted imaging settings for each round of image acquisition, such that a limited number of pixels per field of view was saturated.

The presence of some saturated pixels ensures that the intensity distribution of the sample is mapped to the entire dynamic range of the detectors, while the fact that only a few pixels are saturated suggests that the true brightness of these pixels is underestimated to only a small degree, assuming a smooth brightness distribution.

In practice, we manually adjusted the laser intensity to the lowest values that would allow us to reach this desired degree of saturation (typically 0.5 % for the 633 nm line and 2.5 % for 488 nm) while keeping the required detector gains below 750 to ensure minimal detector noise.

In the confocal imaging workflow, HPFs were acquired in a fully automated and standardized manner along the longitudinal centerline of each microfluidic channel, with an average inter-slice distance of 1.85 μm, resulting in a total Z-depth of approximately 37 μm per field.

Twenty HPFs were collected per channel at predefined spatial intervals, thereby minimizing operator-dependent field selection bias.

By focusing on the different wavelength channels and subsequent segmentation (see below), this enabled precise background removal and enhanced the visualization of morphological cell features (e.g. roundness, area and aspect ratio (the proportional relationship between the width and height of an object)) for the cell recognition and classification training. For the training of the algorithm, we first simplified the categorization of the identifiable transmigration stages (capture, rolling, arrest, intravascular crawling, and paracellular or transcellular transendothelial migration) into morphologically easily distinguishable categories: “attached”, “in process” and “transmigrated” as established for flow-based adhesion assays earlier^24^. The “attached” category encompasses the morphology of cells during capture, rolling, and arrest phases, while “in process” (the initiated transmigration) referred to intravascular crawling, and “transmigrated” represented the paracellular or transcellular transendothelial migration, when cells emerge at the basolateral side of the endothelial monolayer.

To streamline the classification and improve the accuracy of the system, we then adopted a binary approach, merging the “attached” and “in process” stages into a single category (“attached/process”, as afore defined: initiated transmigration), while keeping “transmigrated” as a separate group reflecting a distinct stage within the transmigration cascade (Fig. 1e) referring to cells on the basolateral side of the endothelial monolayer following perfusion.

### Processing of the raw microscopy data

Fluorescence images were acquired, using confocal microscopy, with independent detection channels, producing 16-bit grayscale images per fluorescence channel. For downstream processing, individual channels were exported and converted to grayscale 8-bit TIFF images following background subtraction.

**Fig. 2:**
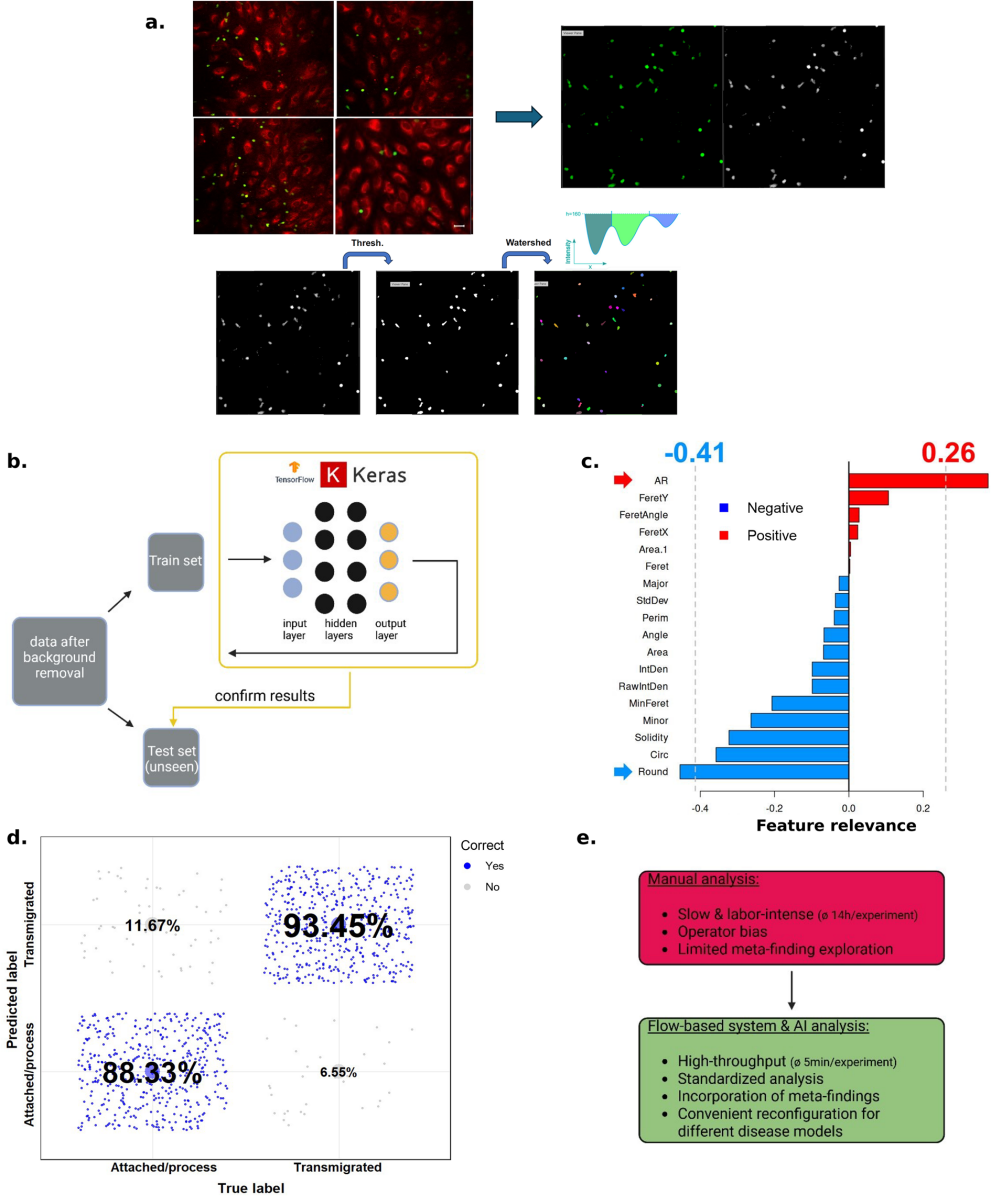
(**a**) Image background removal processing of raw data (confocal microscopy pictures): Red = Endothelial cells (HUVEC); green = T cells. The segmentation of the green signal is achieved through conversion to greyscale (simplification of the color input information through reduction to intensity values) and application of a binary threshold (transformation of complex intensity distributions into a clear, binary representation, in which each pixel is then categorized as either part of the foreground or background signal). Subsequently, post-threshold refinement and watershed segmentation (schematic overview shown, separates overlapping or clustered T cells through delineation of individual cells based on intensity gradients after identification of watershed ridge lines). (**b**) Schematic illustration of the data handling for training and validation of the deep learning model. After processing raw data (confocal microscopy pictures) as shown in a, the model is trained by employing a subset of the whole data set (Train set) and three hidden layers. Afterwards, the model was evaluated in an unbiased approach on the before unseen Test set. Created with BioRender.com. (**c**) Analysis of the feature relevance for the training of the model. Horizontal axis represents the magnitude of feature relevance. Positive relevance shown in red, negative relevance in blue. AR, Aspect Ratio; Feret, Feret Diameter; Major, Major Axis Length; StdDev, Standard Deviation; Perim, Perimeter; IntDen, Integrated Density; RawIntDen, Raw Integrated Density; MinFeret, Minimum Feret Diameter; Minor, Minor Axis Length; Circ, Circularity; Round, Roundness (**d**) The classifier confusion matrix illustrates the performance of the DL model to accurately categorize the extravasation phase of the transmigrating T cells. (**e**) Advantages of new system compared to limitations of the current system relying on manual analysis.

To enhance the extraction of the data in the microscopy pictures, i.e. to identify the transmigrating cells in the picture and enhance their morphological features, we implemented a gradual background removal of the microscopy pictures. The segmentation of the green fluorescence signal (CFSE, T cells) (as shown in Fig. 2a), a central preprocessing step, started with a greyscale conversion of the original image.

Grayscale conversion was applied to simplify feature extraction and reduce computational complexity for the machine learning model. As the classification task relied on morphological features rather than spectral separation or intensity-based quantification, this conversion did not compromise relevant biological information.

This first step simplifies the input by reducing color information to intensity values, as a necessary precursor to thresholding operations. In the greyscale conversion the average pixel values of the primary colors (RGB) are combined. Following this step luminous intensity of each color band is combined into an approximated grayscale value^31^.

The grayscale conversion was followed by the application of a binary threshold, a pivotal measure in segmentation workflows. Herein, this process transforms complex intensity distributions into a clear, binary representation categorizing each pixel as either part of the foreground or background signal^32^. Post-threshold refinement was employed to further enhance the quality of the segmented areas. This refinement is especially crucial in microscopical imaging of biological samples due to their heterogeneity, as more uneven fluorescence signals or image artifacts result in false positives or fragmented cell boundaries, which heavily interfere with downstream analyses^33^.

Afterwards, we employed watershed segmentation on the binary image to separate overlapping or clustered objects, like individual T cells in close spatial proximity. Watershed segmentation is particularly well suited for this task, as it interprets the images intensity landscapes as topographic surfaces, in which high-intensity areas form peaks and low-intensity areas basins (see schematic overview in Fig. 2a). Based on that, the algorithm identified “watershed ridge lines”, which are boundaries tracing the points of steepest decent between adjacent regions hence allowing delineation of individual cells based on intensity gradients. To summarize the preprocessing, it consisted of exporting individual channels and converting them into 8-bit grayscale TIFF images. This was followed by the gradual background (default black and White; B&W) removal (images were adjusted to acquire the main signal channel). The segmentation of the green fluorescence signal was then achieved by converting to greyscale and applying a binary threshold, keeping circularity at 0.10 or higher. This approach enabled the precise definition of cell boundaries and the extraction of crucial cellular features, including Area, standard deviation, X|Y coordinates, Perimeter, Circularity, and Solidity from each segmented region^34^. These features form the basis for downstream analyses.

### Identification and classification of T cells during transendothelial migration

Having established a cellular parameter matrix, we assessed in the first model, whether linear discriminant analysis could effectively classify the three migratory states using supervised between-group analysis (BGA). We observed a strong overlap between groups, with no clear segregation (see Fig. 1c) impeding the training (as discussed above). In the next step, building on our recent use of machine learning models for similar biological questions^35,36^, we applied a Keras/TensorFlow machine learning model (representative workflow Fig. 2b) to employ a widely adopted system for biological data analysis, with a user-friendly, high-level Application Programming Interface (API)^37^. This accessibility enables other researchers, even those without extensive machine learning expertise, to easily adopt our system and build upon it. In the first machine learning model (phase-contrast based microscopy), using a multiclass classifier, we observed, on an unseen validation dataset, high prediction accuracies of 82.1 % and 87.8 % for transmigrated and attached cells, respectively (Supplementary Fig. 1b). In the final analysis, we employed a binary machine learning (ML) model to classify cells as either transmigrated or not (i.e. attached/in process). We then performed a feature extraction analysis to identify cellular parameters (e.g. roundness, area and aspect ratio (see Supplementary Table 1)) which are highly associated with the binary ML model, meaning that these features allow a separation and classification of our cell structures into two distinct groups. Two parameters showed highly relevant associations. The aspect ratio (AR) exhibited the highest positive feature relevance (indicating a higher likelihood of transmigration), while circularity and roundness showed the highest negative feature relevance (Fig. 2c). This model yielded high prediction accuracies of 93.6 % for transmigrated cells and 88.3 % for non-transmigrated cells (“attached/process” (initiated transmigration)) (Fig. 2d) surpassing manual reproducibility rates of 70 %. Furthermore, on average, manual analysis took 10 h per experiment for two (blinded) operators (all 6 channels of microcapillary slide), while the deep learning-based analysis yields results in only minutes.

### AI-powered flow-based adhesion assays for comprehensive studies of TEM: examples from the field

To test suitability and robustness of the model for different research applications we analyzed various samples from healthy donors and different patient cohorts. We sought to analyze as to whether possible variations in morphology due to age, sex and/or disease/prior treatment impact robustness in accuracy. To ensure accuracy we additionally performed a manual re-analysis.

**Fig. 3:**
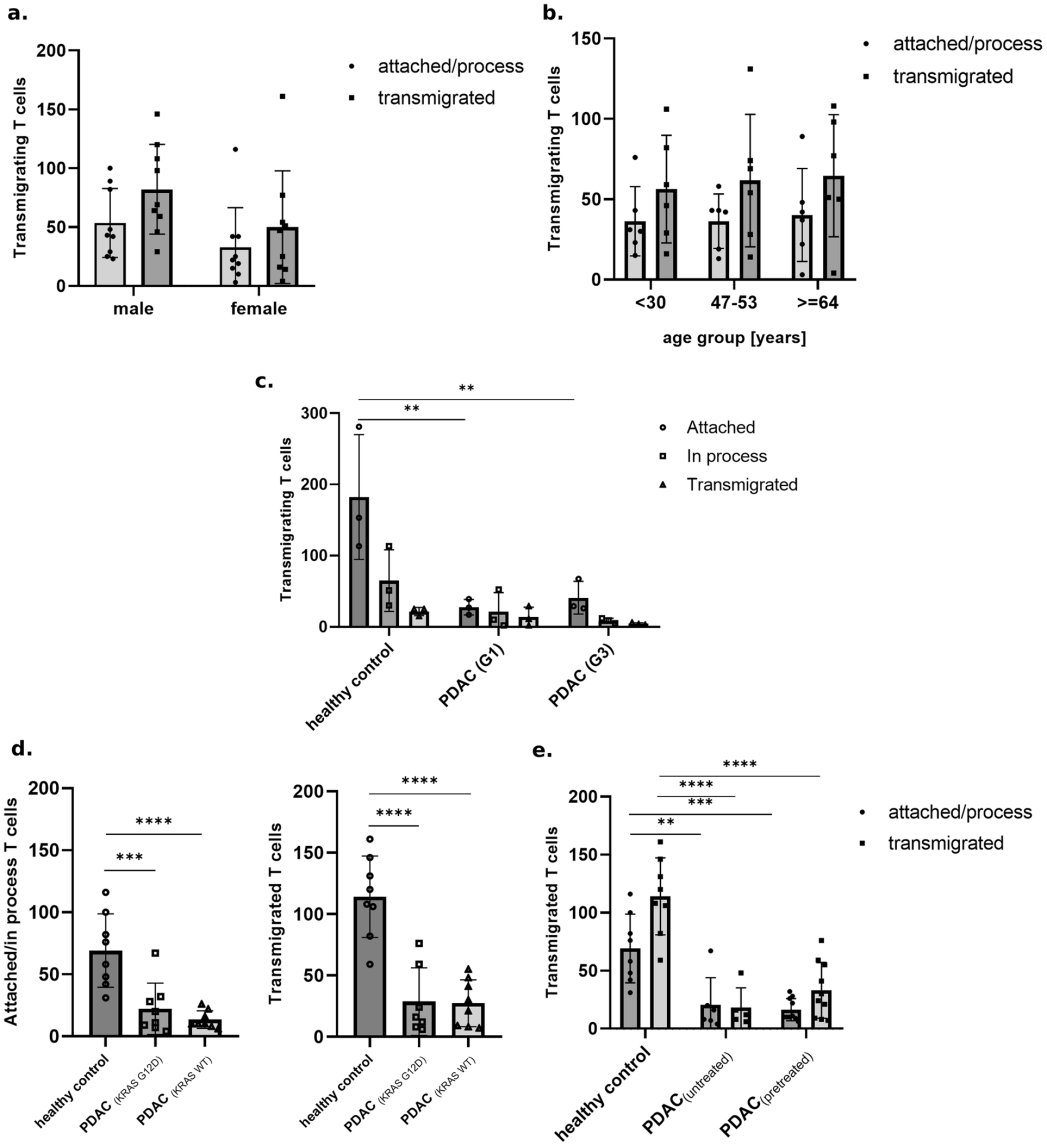
(**a**) Quantification of transmigrating T cells from healthy-donors across age range 18–65 years, comparison between sexes, in the flow-based adhesion assay, employing confocal microscopy, analyzed by Keras/TensorFlow-deep learning system. *N* = 9 biological replicates. Bar heights represent means; error bars represent s.e.m. Transmigration stages: at o process= attached or in process. transmigrated= fully transmigrated. (**b**) Quantification of transmigrating T cells from healthy-donors across three age groups (≤ 30, 47–53 and ≥ 64 years) in the flow-based adhesion assay, employing confocal microscopy, analyzed by Keras/TensorFlow-deep learning system. *N* = 6 biological replicates. Bar heights represent means; error bars represent s.e.m. Transmigration stages: at o process= attached or in process. transmigrated= fully transmigrated. (**c**) Quantification of transmigrating T cells in the flow-adhesion assay, employing brightfield microscopy. Manual assessment and categorization used in early model. *N* = 3 biological replicates. Bar heights represent means; error bars represent s.e.m. P-value was calculated using one-way ANOVA followed by Tukey’s correction for multiple testing. NS, not significant, *P ≤ 0.05, **P ≤ 0.01, ***P ≤ 0.001, ****P ≤ 0.0001. (**d**) Quantification of transmigrating T cells in the flow-based adhesion assay, employing confocal microscopy, analyzed by Keras/TensorFlow-deep learning system. *N* = 8 biological replicates. Bar heights represent means; error bars represent s.e.m. P-value was calculated using one-way ANOVA followed by Tukey’s correction for multiple testing. NS, not significant, *P ≤ 0.05, **P ≤ 0.01, ***P ≤ 0.001, ****P ≤ 0.0001. One outlier was identified and removed using the ROUT test (Q = 1%). The adjusted dataset reflects the exclusion of this value to ensure an accurate representation of the central trend. KRAS variants: wild-type (WT) KRAS; KRAS (G12D). (**e**) Quantification of transmigrating T cells in the flow-based adhesion assay, employing confocal microscopy, analyzed by Keras/TensorFlow-deep learning system. Comparison between healthy donor control and PDAC patient T cells (untreated vs. pretreated). PDAC^WT^ and PDAC^G12D^ samples grouped together by untreated and pretreated. Bar heights represent means; error bars represent s.e.m. P-value was calculated using one-way ANOVA followed by Tukey’s correction for multiple testing. NS, not significant, *P ≤ 0.05, **P ≤ 0.01, ***P ≤ 0.001, ****P ≤ 0.0001. One outlier was identified and removed using the ROUT test (Q = 1%). The adjusted dataset reflects the exclusion of this value to ensure an accurate representation of the central trend. KRAS variants: wild-type (WT) KRAS; KRAS (G12D).

Firstly, we tested if factors like age or different sex had an effect on TEM. Herein, healthy-donor T cells showed no significant differences between male or female donors (Fig. 3a). The extravasation performance (the ability to transmigrate, indicated by the number of cells which are in the phases of transmigration, either the “attached/process” or “transmigrated”, in the standardized experimental time frame in comparison) was also not significantly affected by the donors’ age (comparing age groups: ≤30, 47–53 and ≥ 64 years) (Fig. 3b).

Secondly, we wanted to test T cells from PDAC-patients, as we were, as a secondary objective, interested in their performance, due to PDAC-TME being described as an “immune desert”^12^. Based on results from two patient cohorts (PDAC with tumor grading G1 vs. G3 classified by an independent pathologist) with the initial, phase contrast-based system and manual analysis (Fig. 3c) we sought to investigate whether KRAS mutation leads to changed immune cell transmigration behavior/performance. Patients with KRAS mutations (especially G12D and G12V^15^ have a poorer prognosis due to, inter alia, more aggressively invasive metastasis, changes to the TME and reduced therapy response^16,17^. However, the role of T-cell transmigratory performance is not fully elucidated in this context to date. Therefore, we tested a healthy-donor cohort vs. two patient cohorts with PDAC with KRAS wild-type (KRAS^WT^) and KRAS G12D mutation (KRAS^WT^/KRAS^G12D^).

The extravasation performance of T cells derived from healthy donors significantly exceeded that of PDAC patient-derived cells (shown in Fig. 3d), both in terms of the number of cells in the “attached/in process” phase and the number of fully transmigrated cells. No statistically significant differences were observed between T cells from patients either with the KRAS-mutation or without it (KRAS^G12D^/KRAS^WT^). To eliminate any potential correlation between previous therapeutic intervention and the performance of PDAC-patient-derived T cells, we compared samples sorted by “untreated” and “pretreated” (previous therapeutic intervention: 1st or 2nd line chemotherapy for PDAC) (Fig. 3e).

## Discussion

Transendothelial migration (TEM) is a complex and crucial perquisite for cells traversing endothelial barriers. The goal of our work was to transform a niche-experimental assay for the assessment of this process under flow-conditions/sheer stress, hence closely mimicking *in vivo*-conditions within vascular beddings, into an easily widely-adopt and -adaptable platform for a standardized, timely assessment of cell transmigration in a more physiologically relevant manner.

This tool might enable the advancement of a deeper understanding of both physiological and pathophysiological processes regarding the immune system and the development of new therapeutic interventions in the context of transendothelial migration. Additionally, it offers a very promising approach for assessing the practical application and effectiveness of such therapies in a translational context.

Our deep learning approach enhances the standardization of TEM-analysis, which significantly improves reproducibility and accelerates the process. This automation drastically minimizes the influence of operator bias and reduces the time per experimental analysis from 10 h (average for two operators) to several minutes, thereby enabling fast analyses. Although, it needs to be noted that the current validation is limited to comparison with expert manual annotation.

Also, while conventional high-content imaging approaches are valuable, they require extensive parameter tuning and manual intervention in our dataset, limiting scalability and reproducibility. This motivated the use of a DL-based classifier, which demonstrated improved robustness and substantially reduced analysis time compared to both manual annotation and classical automated pipelines.

By using Keras/TensorFlow, we leverage a widely adopted system for biological data analysis, with a user-friendly, high-level API^37^. This accessibility enables other researchers, even those without extensive machine learning expertise, to easily build upon our work.

Subsequent analysis options, like cluster analysis through Voronoi tessellation (Supplementary Fig. 1c), which includes density estimations and cluster visualization, adds another layer of analytical depth. Moreover, the scope of our method is not limited to T cell analysis but can be adapted and re-trained to analyze other immune cell populations such as monocytes, granulocytes, Natural Killer (NK) or dendritic cells. This also includes the migration performance of non-immune cells, i.e. cancer cells and their metastatic potential^25^. In this context, our tool offers the potential to gain insights into tumor cell extravasation and organotropism, which are key aspects in metastasis. This adaptability promises broad application in diverse research areas, which span oncology, immunology as well as regenerative medicine.

This novel tool could also be applied in a future translational setting to assess organ- or tissue-specific immune cell transmigration, supporting the field of personalized medicine. For this purpose, µ-slides could be seeded with either cell lines closely replicating these tissues or patient-derived primary cells. Herein, researchers could replicate organ- or tissue-specific microenvironments on µ-slide-platforms, which would enable *ex vivo* assessment of TEM under patient-specific conditions.

Although the DL model accounts for variations in data sets robustly, the generalizability of the classifier to different cell types, flow rates, staining intensities, or imaging platforms was not systematically evaluated and represented a limitation of the present work. Furthermore, it must be noted that the deep learning model captures transmigration-associated morphological states under flow, rather than functional transmigration in a fully 3D tissue context.

In the future, this AI-based assay might be further improved by adding complexity for a more advanced, physiological setup, recreating 3D instead of planar 2D structures, achieved through 3D bioprinting more holistic vascular-like channels for further approximation to *in vivo*-blood vessels^38^.

Concerning our results for the performance of PDAC-patient-derived cells, these clearly indicate that the highly significantly reduced transmigration performance, compared to the migration rate of the healthy donor-derived T cells, is not due to previous therapeutic interventions. A possible explanation could be, that it reflects the immune-evasive effects of the tumor-microenvironment (TME) (in this case, of PDAC)^39^. So it could be that the interaction of the T cells with the TME is, independently of therapy or tumor aggressiveness, negatively affecting the TEM performance, limiting their ability to recirculate to other sites of tumor manifestation. The cohort size is rather limited, and the results are therefore of an exploratory nature.

Notably, the model’s high mean prediction accuracy of 91.6 % exceeds the widely accepted 80 % threshold^40,41^. Further improvements in accuracy are unlikely to be benefit-/cost-effective, as the relationship between deep learning accuracy and training size follows a logarithmic trend therefore requiring the disproportionate expansion of the training material from nearly 5 × 10^3^ cells to 5 × 10^4^ in manually annotated cell images. The high accuracy of the system coupled with the inherent standardization of an automated assessment and its fast analysis provides superior results in comparison to the manual analysis, which has several limitations due to operator bias, as well as lower accuracy and longer time-to-result.

Initial accuracy problems of the systems analysis, due to lack of contrast and discrimination of the initial phase contrast recording, were addressed and solved through the introduction of the fluorescence staining and confocal microscopy. This approach allowed convenient background removal through the selection of the wavelength channel. The resulting clear presentation of the transmigrating T cell margins and morphology allowed the training of the system achieve its robustness and precision.

The removal of the Z-stack in the final analysis setup essentially eliminates the aspect of light refractoriness, as used in the original assay. This is supposed to facilitate operator-based assignments to the different phases and was circumvented by the intensity of the fluorescence. Cell morphology, especially the border shape, is clearly distinct through the fluorescence to the background, making categorization easier feasible compared to phase contrast microscopy. Crawling cells potentially prone to detach, cannot be visualized in the static image. Since their amount is within non-relevant percentage-range (≤ 5 %, from our experience in handling the assay), and this should be accounted through the standardization of the assay, this factor is considered negligible.

A factor, becoming relevant after our changes to the original setup, which should be considered, is possible phototoxicity of the fluorescence dye, impacting viability of the T cells^42,43^. All fluorescent dyes were used within the exposure ranges recommended by the manufacturer, and imaging conditions were kept identical across experimental groups.

While no dedicated phototoxicity control experiments were performed, the analysis focused on relative comparisons between conditions acquired under the same imaging parameters. Of note, depending on the nature of dyes employed in future setups, live-cell functional controls might become relevant to ensure that no relevant levels of phototoxicity confound the results.

Furthermore, the flow assay still requires a considerable yield of viable cells (at least 0.5 Mio/mL cell suspension), which is particularly problematic for cells with a low frequency. This could possibly be remedied in the future by using microcapillary slide featuring thinner capillaries. In this context, the high degree of standardization provided by AI is an implicit prerequisite, since manual analysis would likely exhibit excessive fluctuations and the results would be unreliable. Additionally, the current setting does not allow for the simultaneous perfusion of different cell types in a suspension, and thus the subsequent interaction and its impact on TEM. This could be addressed in the future by pre-analytical cell tagging (e.g. fluorescent antibodies), which underscores the advantage of confocal microscopy and could likely be easily achieved by adapting the ML model accordingly.

## Conclusion

Our unique, integrative combination of a flow-based adhesion assay, a so far niche- experimental assay due to lack of scalability and standardization, with the precision of deep learning makes the potential of this powerful tool accessible to a wider audience. This combination now allows ultimate utilization of the advantages that flow assays offer over other live-cell imaging methods in large-scale applications and establishes the necessary standardization required for widespread use. AI can be used to add further add-ins such as Voronoi tessellation. Through spatial and cluster analysis of specific locations of certain cell types, for example, this approach may provide information about their preferred transmigration pathway and thus the use of certain transmigration mechanisms. In a broader sense, our study showed that the combination with AI can leverage seemingly simple and cost-effectively integrated systems to become a very efficient, highly innovative tool.

We are confident that our tool will have a transformative impact on the biomedical research community, drastically enhancing experimental throughput while contributing to the advancement of translational research with direct implications for both clinicians and patients.

## Supplementary Information

Below is the link to the electronic supplementary material.

Supplementary Material 1

## Data Availability

The datasets used during the current study are available from the corresponding author on reasonable request.
