# Supplementary material for: A deep learning approach to assess transendothelial cell trafficking performance
Source: Sci Rep. 2026 Apr 3;16:11602. doi: 10.1038/s41598-026-46045-4 (PMC13057370; doi:10.1038/s41598-026-46045-4)
Supplement: Supplementary file 1 — Supplementary Material 1 [file 41598_2026_46045_MOESM1_ESM.pdf]

A deep learning approach to assess transendothelial cell trafficking performance.

Thomas Michael Schumacher[1], Elisabeth Marie Gottloeber[1], Eric Koziel[1], Mehmet Sacma[6], Kaya Eichhorn[1], Luana Raiber[1], Johann Gout[2], Jessica Lindenmayer[5], Elodie Roger[2], Michael Karl Melzer[2,4], Hartmut Geiger[6], Patrick Christian Hermann[1], Ninel Azoitei[2], Thomas Seufferlein[1], Alexander Kleger[1,2,5,7], Reinhold Schirmbeck[1], Medhanie Assmelash Mulaw[3*] and Yazid Josef Resheq[1*]

[1] Department of Internal Medicine I, Ulm University Hospital, 89081 Ulm, Germany
[2] Institute for Molecular Oncology and Stem Cell Biology, Ulm University Hospital, 89081 Ulm, Germany
[3] Unit for Single-Cell Genomics, Ulm University, 89081 Ulm, Germany
[4] Department of Urology, Ulm University Hospital, 89081 Ulm, Germany
[5] Core Facility Organoids, Ulm University, 89081 Ulm, Germany
[6] Institute of Molecular Medicine, Ulm University, 89081 Ulm, Germany
[7] Division of Interdisciplinary Pancreatology, Department of Internal Medicine 1, Ulm University Hospital, 89081 Ulm, Germany
* M.A.M. (Medhanie.Mulaw@uni-ulm.de) and Y.J.R. (Yazid.Resheq@uniklinik-ulm.de) are co-senior & corresponding authors

Supplementary Figure 1

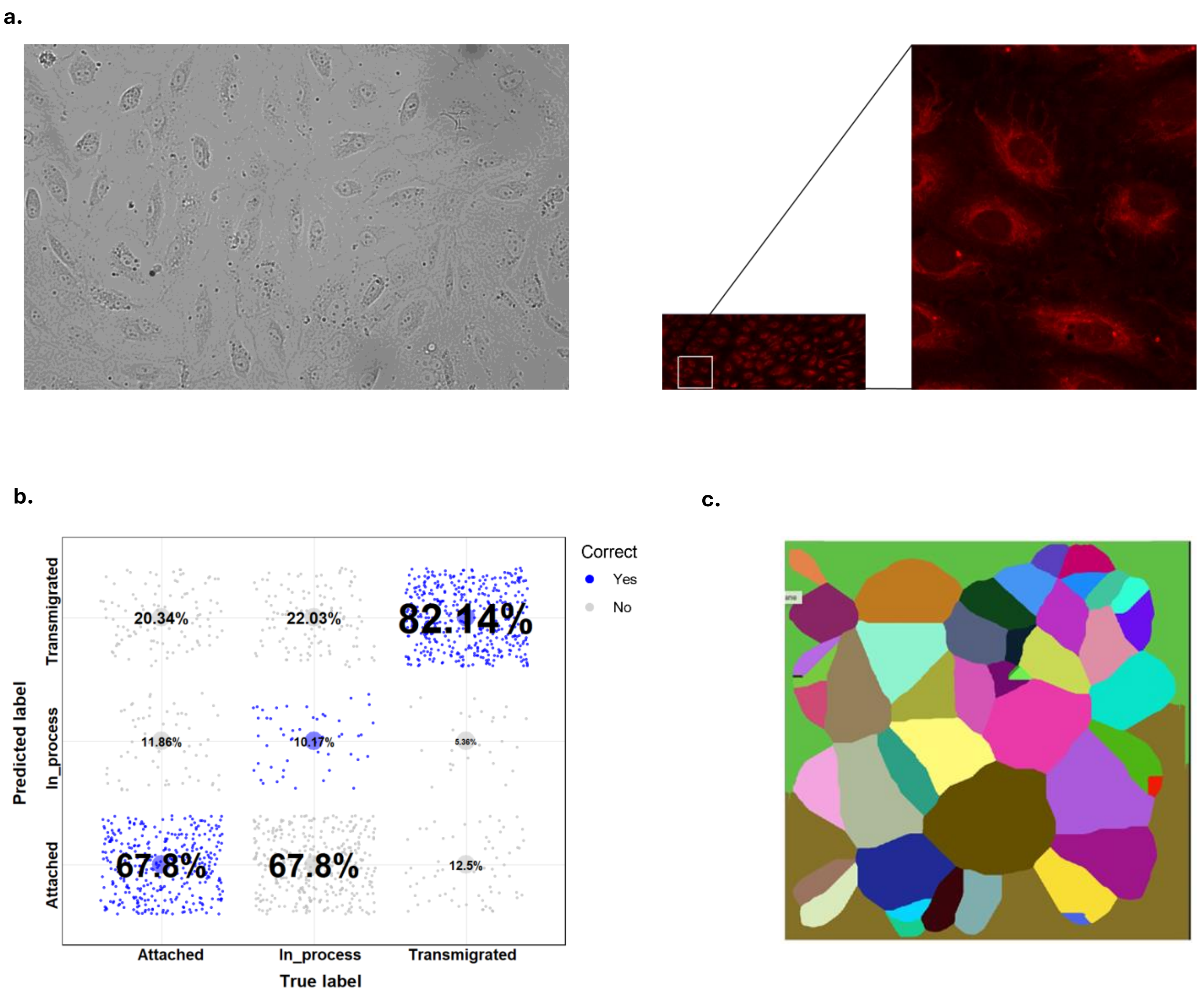

a, Exemplary phase-contrast microscope picture (left) and confocal microscope picture (right; with magnified cutout), under 200x magnification, displaying confluent monolayer. b, The classifier confusion matrix illustrates the performance of the early model to accurately categorize the extravasation phase of the transmigrating T cells. c, Voronoi tessellation (partition of a plane into regions close to each of a given set of objects) of representative image after background removal process; allowing segmentation of cell clustering of transmigrating immune cells.

Supplementary table 1: Geometric feature description

Feature Abbreviation	Full Description	
AR	Aspect Ratio	Ratio of the major axis length to the minor axis length of an object's best-fit ellipse
Feret Angle	Feret Angle	The angle (relative to the x-axis) at which the Feret diameter occurs
Feret	Feret Diameter	The maximum distance between parallel lines tangential to the object
Major	Major Axis Length	Length of the longest axis of the best-fit ellipse that has the same normalized second central moments as the object.
StdDev	Standard Deviation	Standard deviation of pixel intensities within the region of interest (ROI)
Perim	Perimeter	The total length of the boundary (outline) of the object
IntDen	Integrated Densitity	The sum of the pixel values (brightness/intensity) within the selected area (ROI)
RawIntDen	Raw Integrated Density	The direct sum of pixel values, without considering the ROI area or mean
MinFeret	Minimum Feret Diameter	The shortest distance between two parallel tangents touching opposite sides of the object (orthogonal to the Feret angle)
Minor	Minor Axis Length	Length of the shortest axis of the best-fit ellipse around the object
Circ	Circularity	Ratio of the object's area to the square of its perimeter, with a value of 1 indicating a perfect circle
Round	Roundness	Lack of angularity of a shape
